# Synthesis and characterization of kaolin glass cullet ceramics modified with transition metal oxides for enhanced mechanical and optical properties

**DOI:** 10.1038/s41598-025-03908-6

**Published:** 2025-06-02

**Authors:** R. M. Khattab, M. A. Marzouk, H. E. H. Sadek

**Affiliations:** 1 Refractories, Ceramics and Building Materials Department, National Research Centre, Dokki, Cairo, 12622 Egypt; 2 Glass Research Department, National Research Centre, 33 El Bohouth St. (Former El Tahrir St.), Dokki, Giza, 12622 Egypt; 3 Pharos University, Canal El Mahmoudiah Street, Smouha, Alexandria, Egypt

**Keywords:** Ceramics, Kaolin, Glass, Cullet, Waste, Mechanical properties, Chemistry, Materials science

## Abstract

A range of ceramic materials was developed using Egyptian Kaolin combined with varying amounts of glass cullet waste (0–50 wt%) through uniaxial pressing and sintering at temperatures between 900 and 1200 °C. The study further examined the effects of adding transition metal oxides, Co_3_O_4_ or CuO, into a mix of 70% kaolin and 30% cullet, sintered at 1000 °C. Phase identification and chemical composition analysis were carried out using X-ray diffraction (XRD) and dispersive X-ray fluorescence (XRF), while physical properties such as bulk density, apparent porosity, hardness, and microstructure were evaluated through scanning electron microscopy (SEM). The results revealed that increasing the cullet content up to 50 wt% resulted in higher apparent porosity. The sintered ceramics exhibited a hardness of 7.9 GPa, with the lowest bulk density (2.75 g/cm^3^) and highest apparent porosity (13%). Adding Co_3_O_4_ or CuO up to 30 wt% increased the density of the material and reduced porosity, with Co_3_O_4_ achieving the highest density (2.44 g/cm^3^) and lowest porosity (13%). CuO slightly increased porosity to around 4%, with a density of 2.46 g/cm^3^. Co_3_O_4_-based ceramics exhibited superior hardness compared to CuO, as the latter encouraged the formation of anorthite. Optical tests showed that Co_3_O_4_ caused a color change from light to dark, while CuO samples turned dark brown to black. CuO-containing ceramics had reflectance values below 40%, indicating their potential application in antireflection coatings for solar cells.

## Introduction

Municipal solid waste (MSW) disposal issues have drawn a lot of attention, particularly from an environmental perspective. Therefore, it is almost welcome to engage in any activity that would facilitate its reduction and valorization^1,2^. Demands for more rigorous recycling efforts are unavoidably a result of pollution issues. Reduced sintering temperature^3,4^, lower shrinkage during the drying and sintering processes^3,5^, and decreased water absorption of the ceramics^3,6^ are some advantages of using glass waste in clay ceramic products. Many nations have started to restrict brick manufacturing in response to the global scarcity of natural clay and have been looking for other building materials. As a result, there are a lot of more connected studies into the production of clay bricks from different sources. A promising method to reduce the amount of natural clay used in the brick-making industry is the partial substitution of waste materials for clay, such as organic solid wastes, construction and demolition wastes, agricultural solid wastes, plastic wastes, and industrial wastes^7–17^.

Cullet makes a significant contribution to the collection of municipal solid waste (MSW), per reports^1–18^. This cullet can be utilized as a fluxing agent in the manufacturing of ceramics, including stoneware, tiles, bricks, concrete, and cement, as other studies have shown^19,20^. When combined with clay, it can also lower the sintering temperature^21^. Depending on its intended purpose, leftover glass that has been crushed into a variety of particle sizes, from coarse aggregates to extremely fine powder, is referred to as cullet^22^. Cullet is currently one of the main waste streams that are becoming a serious environmental problem due to the recent massive global consumption^7,23,24^. Glass cullet waste (GCW) can be recycled to create new goods, but because of impurities and the high expense of the recycling process, a large amount of GCW may end up in landfills^7,25^. Approximately 30% of GCW is thought to be disposed of in landfills worldwide^7,26^. The Environmental Protection Agency (EPA) estimates that GCW makes up 4.2% of all trash collected, with 61.7% of that amount ending up in landfills^7,27^. Glass pharmaceutical packaging, residential and commercial construction sites, and beverage firms are the sources of these pollutants. Because the cullet is rich in SiO_2_, CaO, Na_2_O, and Al_2_O_3_, it seems to be a good raw material for ceramic bodies^22^. Another way to use this waste is in ceramics. Known as soda-lime-silica composition, sodium oxide, calcium oxide, and silicon dioxide makeup almost 95% of all manufactured glass. Because of its low softening temperature, cullet is frequently employed as an additive in glass reforming^22,28^. Cullet constitutes a serious nuisance to the environment, and a burden to humans since it is inert, not biodegradable, and very difficult to dispose of as it can remain active in a dumpsite for a thousand years. Cullet is used as road construction aggregate, asphalt paving, concrete aggregate, and building applications (glass tiles, bricks, wall panels, etc.)^22^.

On the other hand, the clay material called kaolin is frequently used for paper coating and filling^29^. Al_2_Si_2_O_5_(OH)_4_ is a unique phyllosilicate mineral with this chemical formula. In the 1:1 di-octahedral structure of kaolin clay, one octahedral Al_2_(OH)_4_ alumina plate is connected to one tetrahedral SiO_4_ silica by an oxygen atom^29^. Because of their sufficient mechanical, durability, and thermal performance, clay bricks are widely utilized worldwide^7,30^. The primary factors influencing the characteristics of clay bricks are typically the raw ingredients, production procedures, and firing temperature. However, high-performance burned brick is necessary to ensure long serviceability in the contemporary construction sector. The fusing phase of silica and alumina elements in clay has produced a ceramic-like bond and uniform fine pores, which are meant to make bricks strong^7,31^. Particles of clay fuse and form bonds with one another when they are burned at high temperatures. Therefore, clay bricks can be treated with additives such as fluxing agents (inorganic wastes) to improve the bond at low temperatures^7,32^. Around the world, several materials have been utilized to enhance the sintering qualities of clay brick, including fly ash, glass, marble residue, arc furnace steel dust, calcium carbonate sludge (a byproduct of the stone industry), paper mill sludge, and sewage sludge^7,33^. The inorganic additives strengthen the link between bricks, boost particle adhesion, and stabilize clay during burning. Consequently, a brick with a consistent microstructure and high strength is created.

It has been discovered that adding glass increases manufacturing capacity by lowering fire temperature and time^22,34^. These novel ceramics (clay-cullet) have been demonstrated to have improved density, hardness, drying shrinkage, water absorption, and other favorable physical properties^1,19,22^.

Recent studies^35–37^ demonstrate that cullet recycling reduces energy consumption by 32–38% in glass production compared to virgin materials, while each ton of cullet avoids about 0.7 tons of CO₂ emissions through raw material substitution and reduced calcination^35–37^. However, challenges persist in post-consumer cullet utilization, particularly in architectural glass, where strict color sorting and quality standards limit recycling rates. Innovations in mixed-color cullet processing now enable its viable use in non-aesthetic applications, such as structural ceramics, bypassing traditional color constraints^35–37^. Recent work^37^ on kaolin-polymer composites reveals that kaolin content > 30 wt% significantly improves thermal stability, delaying decomposition by ~ 50 °C, suggesting similar synergistic effects may occur in glass-kaolin systems.

The main objective of the current study is to synthesize and characterize a series of ceramic composites by integrating Egyptian kaolin with recycled glass cullet waste (0–50 wt%), processed via uniaxial pressing and sintered across different temperature gradient of 900–1200 °C, to evaluate the influence of waste incorporation and thermal treatment on microstructure, mechanical properties, and sustainability potential. Also the study aimed at Optimizing heat treatment for employing a novel approach that not only supports environmental responsibility through waste reduction but also examines the thermal and mechanical advantages provided by the glass phase. Characterization of glass–ceramics was done using density, porosity, XRD, SEM, and reflectance measurements.

Moreover, this approach is unprecedented in Egypt, as no prior research has documented the integration of cullet into ceramic recipes within the region. Our investigation extended to focus on how different firing temperatures influence the mechanical, structural, and physical properties of the final ceramic products. After determining a composition that exhibits desirable technological characteristics, we further examined the effects of varying amounts of cobalt and copper oxides on this formulation. The goal is to create colored ceramic bodies suitable for a wide range of applications, including colored bricks, pigments for ceramic and enamel glazes, as well as uses in paper, paints, catalysts or photocatalysts, and sensing technologies^38–44^. The current research underscores the potential of recycled materials in ceramics and creates new opportunities for sustainable practices in material manufacturing.

## Materials and experimental method

### Starting materials

The cullet was collected from trash sites, alcoholic beverage establishments, restaurants, and pharmacies. The Sinai Manganese Company was the source of the kaolin raw materials. Co_3_O_4_ and CuO, respectively, are purchased from SRL and Spectrum with purity of 99%. Table 1 displays the results of a quantitative analysis of the chemical compositions of the kaolin and cullet utilized in the study using wavelength dispersive X-ray fluorescence (AXIOS, WD-XRF Sequential Spectrometer (Panalytical, 2005). XRF of Kaolin indicated that mainly alumina and silica are presented with some impurities like Fe_2_O_3_ and TiO_2_ in the presence of other oxides.

**Table 1:** XRF analysis of the raw materials.

Oxide type	Content in wt%	
	Kaolin	Waste glass
SiO_2_	50.01	71.4
TiO_2_	1.57	0.41
Al_2_O_3_	32.87	7.19
Fe_2_O_3_	1.54	0.38
MgO	–	0.64
CaO	0.24	12.23
Na_2_O	0.22	7.46
K_2_O	–	0.05
P_2_O_5_	0.15	0.02
SO_3_	0.39	0.07
Cl	0.09	–
LOI	12.59	–

### Experimental method

A proportional amount of cullet and Kaolin clay of 100μm in size are mixed according to the composition given in Table 2. In the mixture composition, the varying ratio of cullet on the expense of kaolin is prepared (0, 10, 20, 30, 40, and 50 wt% cullet) and are labeled; K, KC1, KC2, KC3, KC4, and KC5. The mixtures are then homogenized using ball milling for 1 h. The obtained mixtures are subjected to press at 25 MPa followed by drying at 100 °C for 24 h at room temperature. Then it is heat treated at 950, 1000, 1100, and 1200 °C for 2 h to get the ceramic samples finally. Subsequently, a composition with optimal physical and mechanical properties is selected from the mixture, and different concentrations of cobalt oxide or copper oxide are incorporated (10, 20, and 30 wt%) as seen in Table 3 and sintered at 1000 °C to study the effect of adding transition metal oxides on the production of a colored ceramic product with superior technological properties compared to the parent composition. The final prepared samples are designated by incorporating transition metal oxides, such as Co10, Co20, Co30, Cu10, Cu20, and Cu30.

**Table 2:** Chemical composition of the prepared samples in wt. %

Sample No	Kaolin	Waste glass
K	100	0
KC1	90	10
KC2	80	20
KC3	70	30
KC4	60	40
KC5	50	50

**Table 3:** Chemical composition of the prepared transition metal doped samples in wt%

Sample No	Kaolin	Waste glass	Co_3_O_4_	CuO
Co10	70	30	10	–
Co20	70	30	20	–
Co30	70	30	30	–
Cu10	70	30	–	10
Cu20	70	30	–	20
Cu30	70	30	–	30

### Characterizations

The phase compositions at different sintering temperatures were examined using the X-ray diffraction (XRD) technique with monochromatic Cu-Kα X-ray radiation (λ = 1.54 Å), Model (D 500, Siemens, Mannheim, Germany) and a scanning rate of 2 degrees per minute. Using FEI, QUANTA FEG, 250, a scanning electron microscopy (SEM) analysis was performed to ascertain the morphology of the sintered samples. The Archimedes water displacement method was used to determine the apparent porosity and bulk density following the American Society for Testing and Materials (ASTM C373-88)^45^ recommendations.

Three test samples are saturated with water and then fired for two hours. The saturated samples were weighed once more in the air (W_s_) following their water immersion (W_I_). The samples were dry-weighted (W_d_) after being kept at 110 °C for the entire night. The following formulas (1 and 2) were used to calculate the test samples’ apparent porosity (AP) and bulk density (BD):

1 $${\text{AP}} = \frac{{\left( {W_{S} - W_{d} } \right)}}{{\left( {W_{S} - W_{I} } \right)}} \times 100$$

2 $$BD = \frac{{W_{d} }}{{\left( {E_{S} - W_{I} } \right)}} \times \rho$$

where *ρ* is the density of the immersed medium.

A hardness testing machine was used to prepare the samples and assess their hardness by ASTM Standard C730-98. Every sample that was examined had a diameter of 10 mm. During testing, indentations were created under various loads, ranging from 100 to 2000 mN. Since these stresses were applied at a constant pace of 1 mm/s, each indentation was allowed 16 s to equilibrate before the measurement was taken. The microhardness (H), expressed in GPa at the specified velocity, was then calculated using a preset equation^46^:

3 $${\text{H}} = {1}.{854}\left( {{\text{P}}/{\text{d}}^{{2}} } \right)$$

where d is the diagonal of the impression and P is the applied load on the sample.

Lastly, a Cary 300 Bio Varian UV–Vis spectrophotometer was used to perform diffuse reflection spectroscopy (DRS) at room temperature in the 190–2500 nm range with a D65 illuminant and a standard observer angle of 10. It is carried out to ascertain how color behavior affects wavelength spectra region and reflectance band intensities. The compressive strength of the fired specimens was determined by the hydraulic testing machine; Tinius Olsen Universal multi testing machine (UK) model 25ST, with crosshead speed of 0.5 mm/min. CCS was evaluated according to ASTM C 1424-19.

## Results and discussion

Table 1 shows the XRF of the starting materials (kaolin and cullet). The presence of Na_2_O (alkali oxide) and CaO (alkaline earth oxide) in the cullet composition indicates the presence of strong fluxing oxides, which are Na_2_O, and CaO. The main oxides in the cullet are SiO_2_, CaO, and Na_2_O. CaO is a secondary mid-temperature flux that is always introduced through the addition of lime, also known as whiting (CaCO_3_), calspar, also known as anorthite (CaO.Al_2_O_3_.4SiO_2_), or dolomite (MgCO_3_.CaCO_3_) to the recipe during formulation^22^. In the case of kaolin, the main components were silicon and aluminum oxides; in addition to some minor oxides as seen in Table 1.

### Studying the effect of the addition of various ratios of cullet on kaolin

#### Apparent porosity and bulk density

The porosity of brick significantly affects its performance and quality. Brick’s porosity is caused by a few small capillaries. Because of this capillary action, brick transports moisture ten times faster than other building materials. At low temperatures, moisture is reabsorbed, while at high temperatures, it is released from the brick’s pores^47^. As a result, bricks control both the interior and external temperatures of the building. However, high porosity exposes burned clay bricks to chemical attacks from sulfate and salt as well as damaging weathering processes like rain and air pollution. Porosity is associated with dehydroxylation processes, carbonate breakdown, water evaporation, and biomass residual combustion^7,48–50^.

**Fig. 1:**
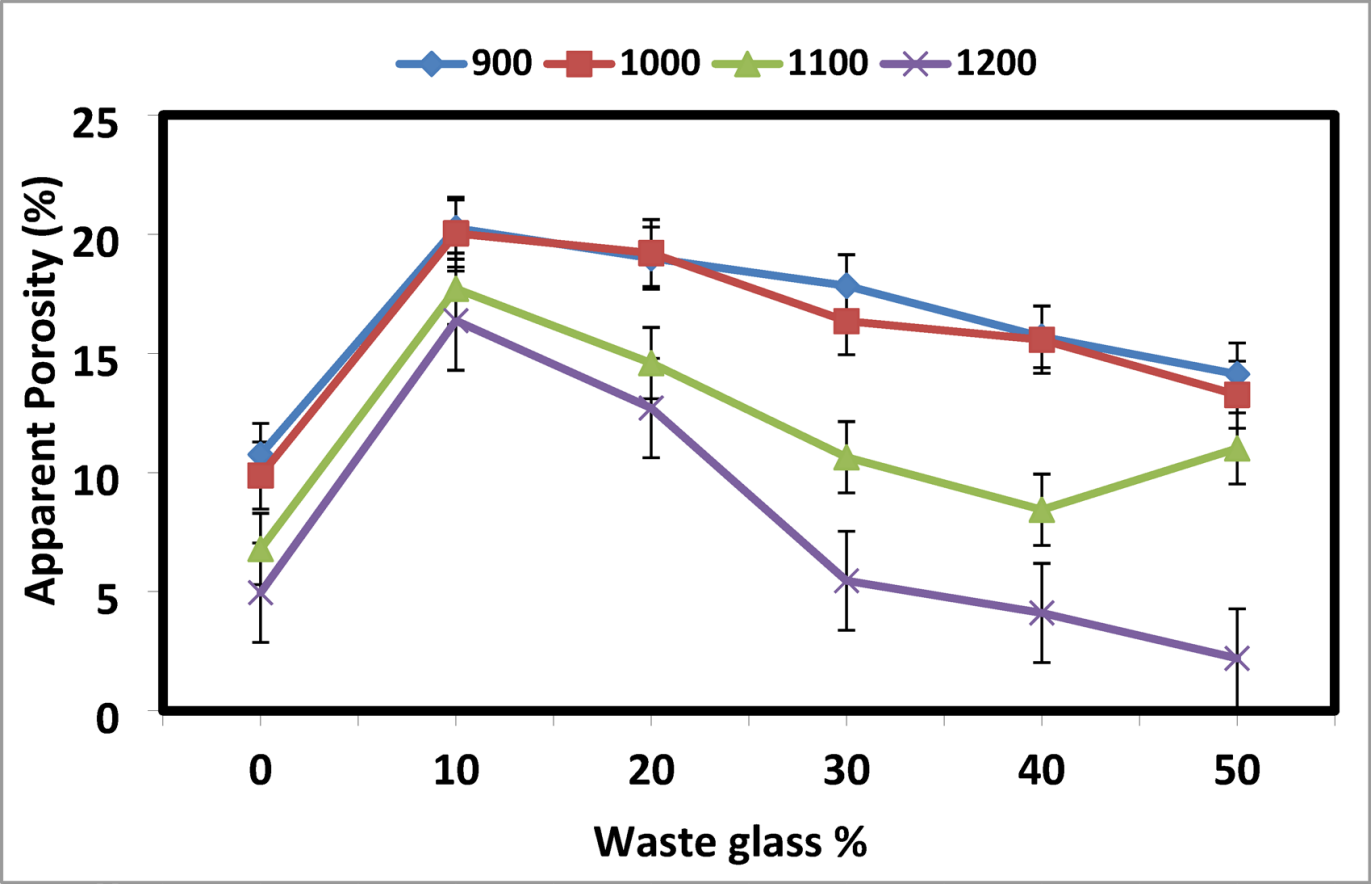
The apparent porosity of the prepared ceramics from mixed kaolin/waste cullet at different sintering temperatures.

**Fig. 2:**
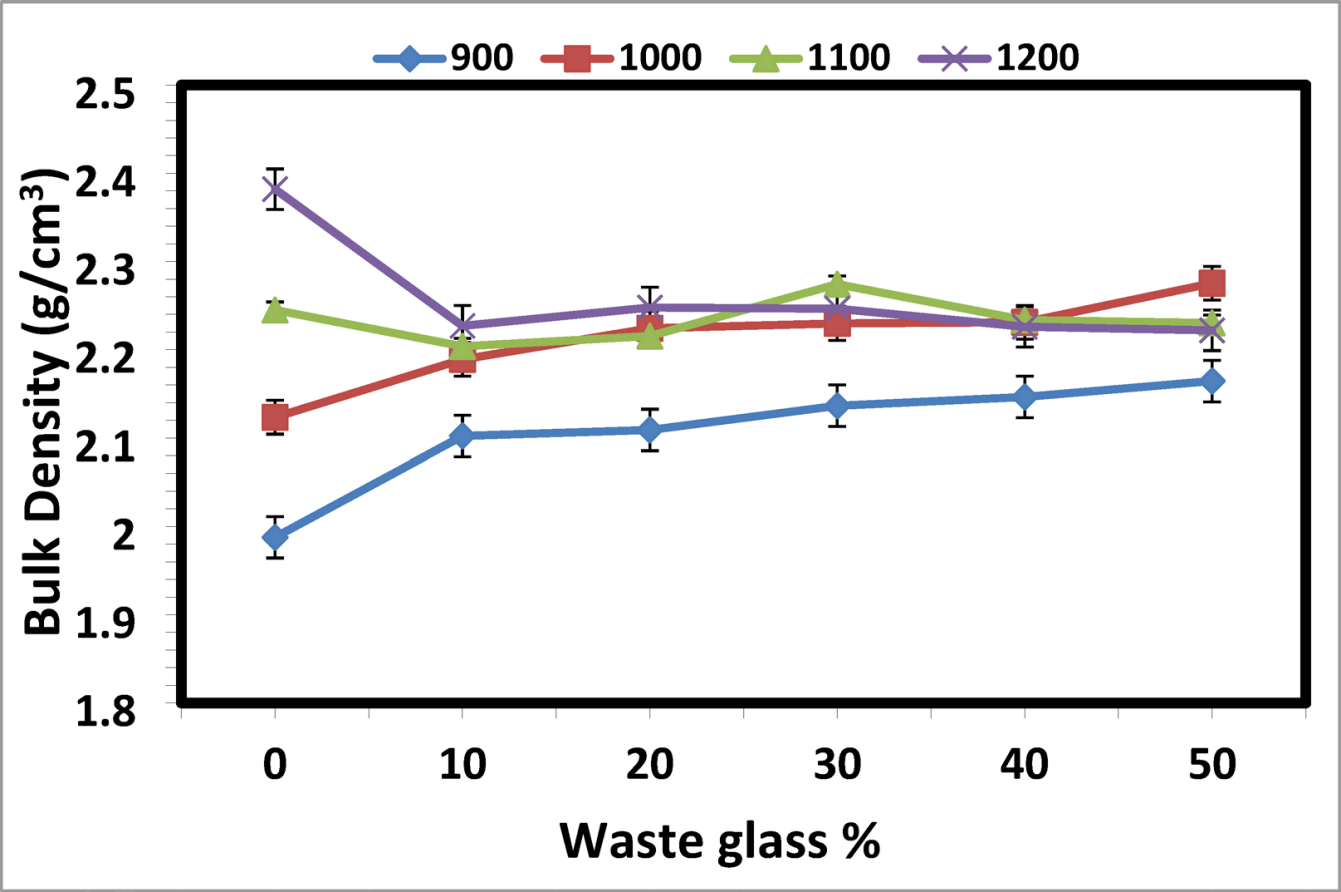
The bulk density of the prepared ceramics from mixed kaolin/waste cullet at different sintering temperatures.

Bricks typically develop tiny pores as a result of the breakdown of organic substances^7,51^. The brick specimen’s apparent porosity and bulk density values following sintering at different temperatures (900, 1000, 1100, and 1200 °C) are displayed in Figs. 1 and 2. The result reveals that the increasing amount of cullet in kaolin and sintering temperatures up to 1200 °C resulted in the least porosity of the samples and rise in density. Sample with 50 wt% addition of cullet and sintering at 1000 °C, give a maximum density and minimum porosity with 2.75 g/cm^3^ and 13.2%, respectively.

Increasing the sintering temperature to 1100 °C, after adding 40 and 50 wt% of the cullet, causes a decrease in bulk density and apparent porosity. After adding 30 wt% of the cullet, the density decreases when the sintering temperature is raised to 1200 °C. Increased fluxing oxides in the body due to a higher cullet content cause the body’s quartz and kaolin to melt and flow even at moderate temperatures. The bulk density rises at 1000 °C as a result of this flow’s tendency to fill and seal all gaps.

Therefore, a higher cullet is associated with a bigger buildup of fluxing oxides in the body and a lower verification temperature. However, the abrupt drop in density following the addition of 40 wt% cullet at 1100 °C and 30 wt% cullet sintered at 1200 °C could be caused by a significant rise in vitreous phase, which causes SiO_2_ and Al_2_O_3_ to transition into liquid phase at higher temperatures^1^.

Many previous studies^52–55^ have confirmed that the phase separation dynamics are further governed by oxide content and thermal treatment. Regarding the results of the XRF analysis in Table 1, minor nucleation oxides like TiO₂ and CaO significantly influence phase separation and crystallization in ceramic materials by altering thermodynamic stability, viscosity, and nucleation kinetics^52,53^. TiO₂ acts as a nucleating agent, promoting liquid–liquid phase separation through coordination changes (e.g., Ti^4^⁺ transitioning from fourfold to sixfold coordination during heating), which initiates nanocrystalline TiO₂ formation and enhances heterogeneous nucleation^52,53^. This reduces crystallization temperatures and activation energy, enabling finer crystal distribution and denser microstructures. While CaO modifies liquid-phase viscosity, facilitating mass transfer and densification during sintering^54,55^. When combined with TiO₂, it lowers viscosity to promote particle rearrangement, while TiO₂ stabilizes phase-separated droplets and catalyzes crystallization of high-aspect-ratio crystals^55^.

Overall, it was found that the cullet samples containing kaolin had more holes than the pure kaolin samples. This is because some CaO is present in the kaolin and cullet. X-ray fluorescence (XRF) analysis revealed that the loss on ignition (LOI) values in Egyptian kaolin samples reach up to 12.59 wt%, a phenomenon attributed to the release of volatile gases during heating^56^. This gas evolution is likely associated with the thermal decomposition of carbonate mineral impurities, such as calcite (CaCO₃) or dolomite (MgCa(CO₃)₂), which may naturally occur in trace amounts within the kaolin structure^56^. As the carbonates decompose thermally, they release carbon dioxide (CO₂) and other gases, leaving behind voids that contribute to the development of a porous microstructure.

A considerable amount of CO_2_ is released during the breakdown of this calcium oxide (CaO). Hence, the apparent porosity rises due to the volatile nature of CaO^7^.

#### XRD analysis of kaolin-cullet samples

**Fig. 3:**
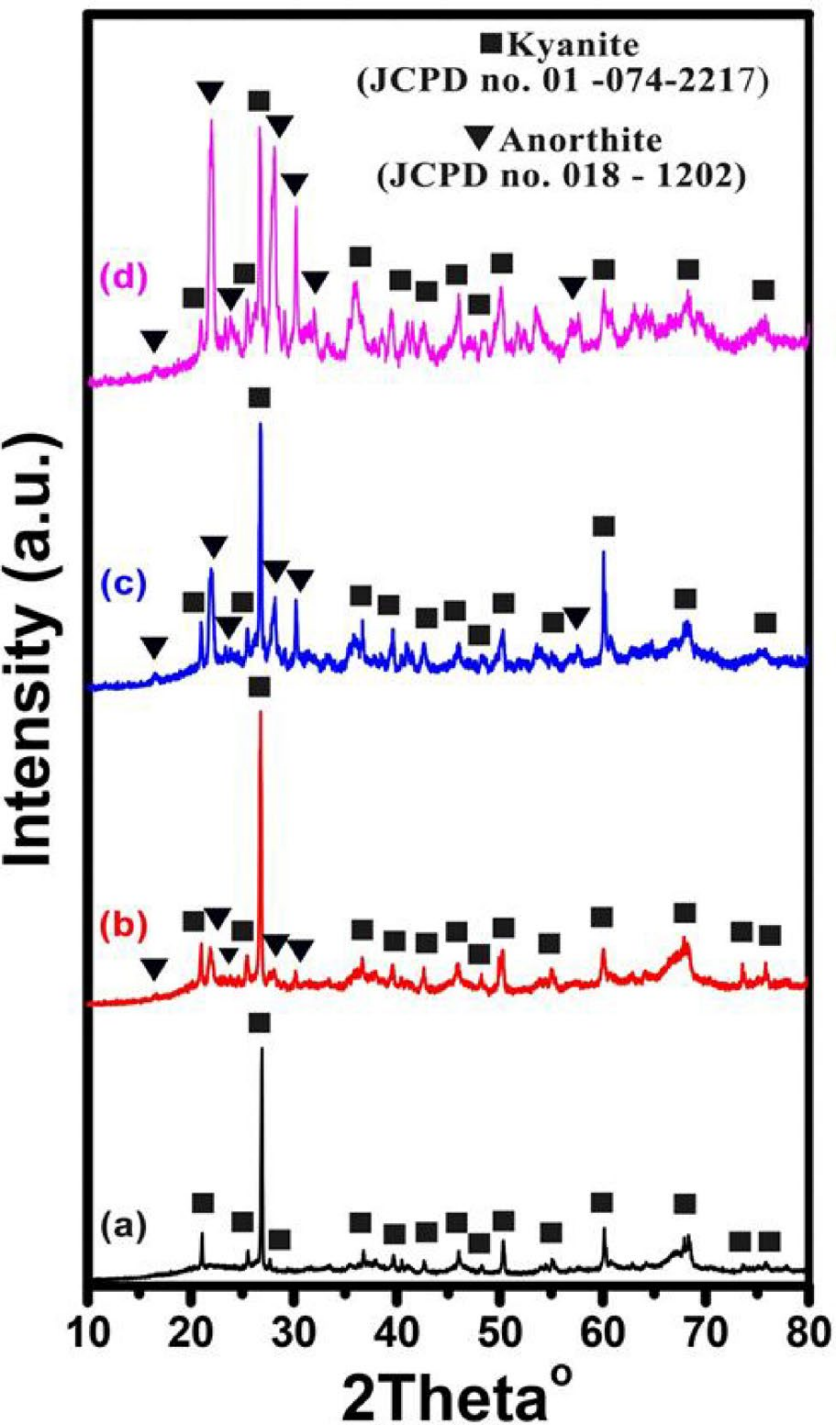
X-ray diffraction of the selected samples from the first series sintered at 1000 °C where (**a**) 0, (**b**) 10, (**c**) 30, and (**d**) 50 wt% of glass cullet waste.

**Fig. 4:**
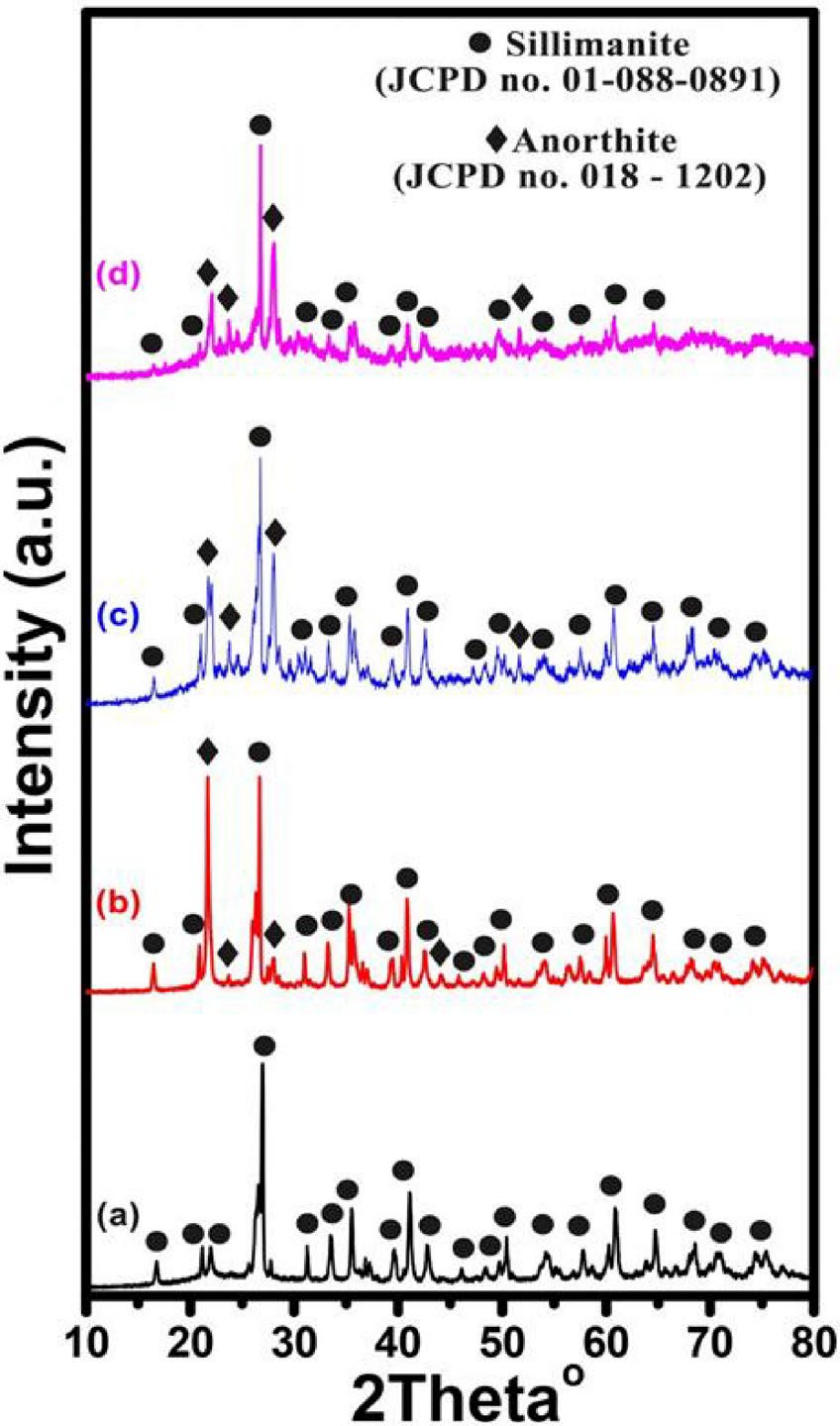
X-ray diffraction of the selected samples from the first series sintered at 1200 °C where (**a**) 0, (**b**) 10, (**c**) 30, and (**d**) 50 wt% of glass cullet waste.

A typical XRD pattern of a few chosen sintered samples is displayed in Figs. 3 and 4 for samples K, KC10, KC30, and KC50 which are sintered at 1000° and 1200° C, respectively, and are shown in Figs. 3a and b. To identify minerals, the acquired XRD patterns were compared to information supplied by the International Center for Diffraction Data (ICDD), formerly known as the Joint Committee on Powder Diffraction Standards (JCPDS)^51,57^. Kyanite (Al_2_SiO_5_) and anorthite (Ca(Al_2_SiO_8_)) were found in the samples that were sintered at 1000 °C, according to the study. The formation of sillimanite (Al_2_SiO_5_) and anorthite occurs when the sintering temperature is raised to 1200 °C as seen in Fig. 4. These phases are typically found in cement, ceramics, and glass–ceramic materials made from glass powder and burned waste^58–60^. The K sample exhibits a kynaite phase at 1000 °C, as seen in Figs. 3. Sillimanite structure appears when the sintering temperature is raised to 1200 °C. The quantities of anorthite at 1000 °C and 1200 °C grow as the cullet addition increases. This results from the addition of a cullet, which raises the ratio of SiO_2_, CaO, and Al_2_O_3_. Generally speaking, mineralogy is aware of the significance of the three Al_2_SiO_5_ aluminosilicates: sillimanite, andalusite, and kyanite. One characteristic of the crystal structure that unites the three polymorphs is the presence of chains of AlO_6_ edge-shared octahedra parallel to the crystallographic b-axis. Kyanite has a triclinic unit cell, whereas sillimanite and andalusite contain orthorhombic unit cells. The principal coordination of one of the aluminum atoms, which is in tetrahedral coordination in sillimanite, five coordinated in andalusite, and octahedral coordination in kyanite, changes amongst the three Al_2_SiO_5_ polymorphs^61^.

#### SEM analysis of kaolin-cullet samples

**Fig. 5:**
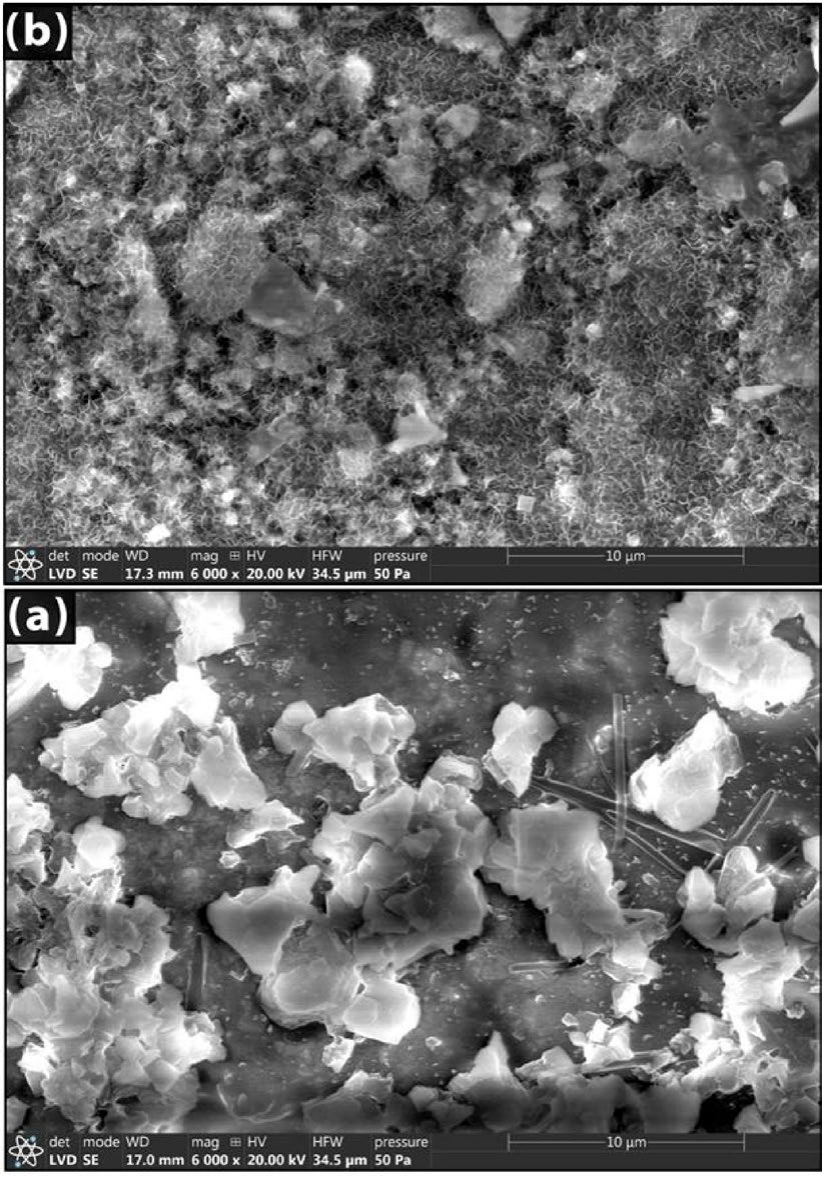
SEM images of samples with 30 wt% (**a**) and 50 wt% (**b**) of cullet that were sintered at a temperature of 1200 °C.

The SEM images of chosen samples with 30 and 50 wt% of cullet that were sintered at 1200 °C are displayed in Fig. 5. Increasing the cullet percentage was found to increase the amount of crystallization. Particles of a needle seem to be fully lodged in the ceramic matrix. Examining the surface morphology in greater detail reveals that a higher cullet enhances the likelihood of uniformly dispersed grains, a high contact surface, and a subsequent decrease in porosity. As cullet addition increases, a larger degree of vitreous phase with a smoother texture is seen^1^.

The SEM scans show no signs of cracking. Because of its nearly non-plastic nature, the fragmented glass that was added to the plastic clay-water mixture served as a degreaser, reducing internal body humidity and, as a result, drying shrinkage^1,62^. As illustrated in Fig. 5, according to the effect shown, there is less chance of drying brought on by breakage and segregation (i.e., cracking) inside the clay-containing glass.

#### Hardness of kaolin-cullet samples

**Fig. 6:**
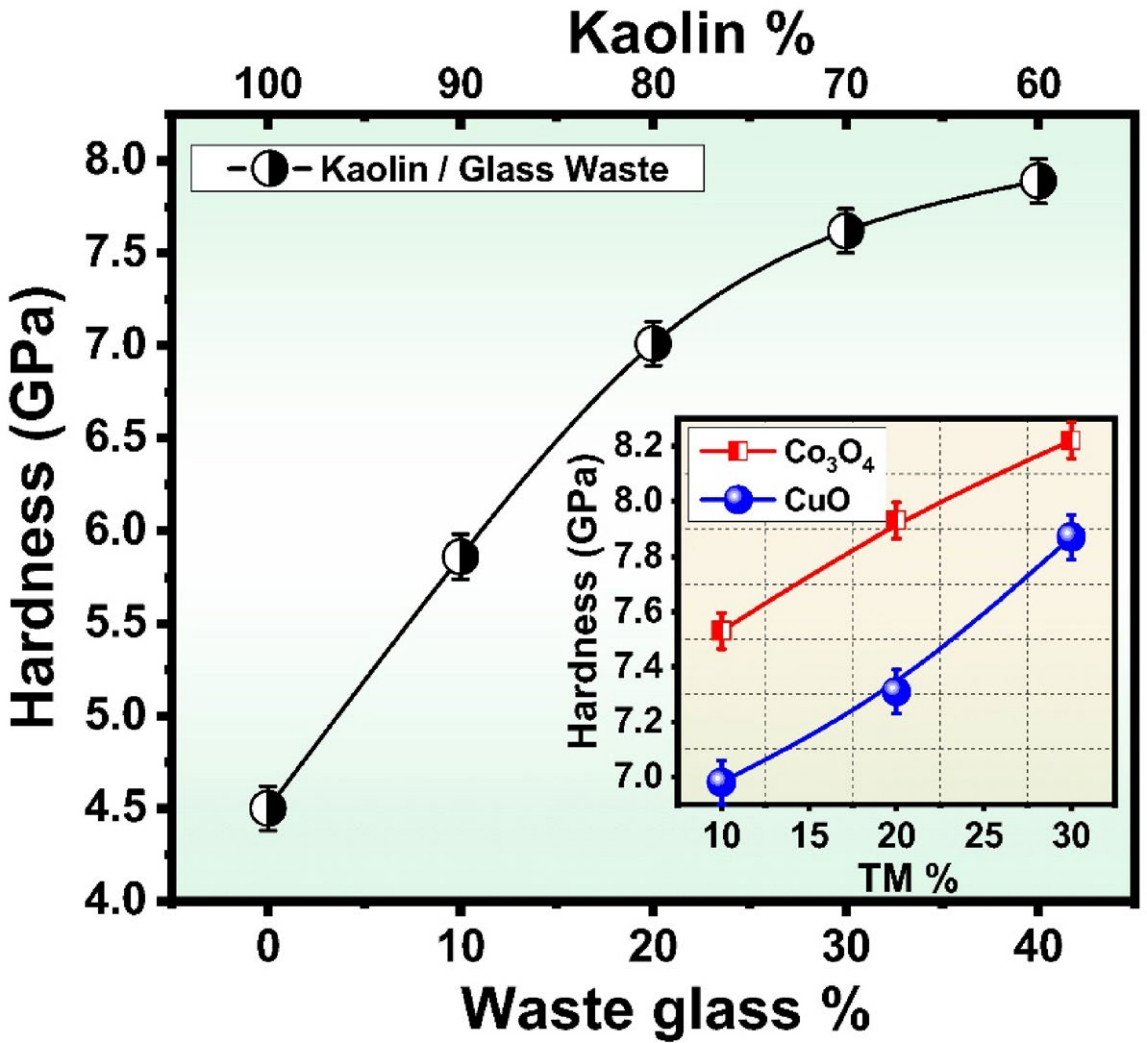
The hardness behavior of the prepared samples.

The mechanical properties in terms of hardness are measured for all samples sintered at 1000 °C as seen in Fig. 6 and their values are listed in Table 4. It is observed that the hardness of samples without glass is 4.5 Gpa. This is due to the lowest density of this sample compared with other samples with cullet.

**Table 4:** The average hardness of some selected samples fired at 1000 °C.

Sample	K	KC1	KC2	KC3	KC4	KC5	10Co	20Co	30Co	10Cu	20Cu	30Cu
	± 0.12 (GPa)						± 0.065 (GPa)			± 0.08 (GPa)		
Hardness (GPa)	4.5	4.93	5.86	7.01	7.62	7.89	7.53	7.93	8.22	6.98	7.31	7.87

The hardness of ceramic materials is closely linked to their density^63^, with higher density leading to increased hardness due to a reduction in porosity and an improvement in structural integrity. As the density of a ceramic increases, the number of voids and defects within its microstructure decreases, which in turn reduces the number of stress concentration points where cracks are likely to initiate and propagate. This relationship between density and hardness arises because denser ceramics have fewer pores and defects, which act as weak points that can compromise the material’s mechanical strength. By minimizing these flaws through enhanced sintering processes or optimized material composition, the resulting ceramic exhibits improved resistance to deformation and fracture, making it more suitable for demanding applications where high mechanical performance is required. On the other hand, the study by Kumar et al.^64^ depicted other reasons that contributed to the increase in hardness. The increase in hardness is attributed to the reduction of non-bridging oxygens (NBOs), which results in a more compact glass network. This is clearly reflected in both the structural and physical properties of the glasses^64^. There is a direct correlation between hardness and the presence of NBOs in the glass structure. NBOs weaken the glass network by decreasing the interconnectivity, which allows the indenter to penetrate further into the sample under a given load. Consequently, as the NBOs increase, the hardness of the glass decreases due to the greater penetration depth.

Figure 6 illustrates the hardness values of fired ceramic samples subjected to a temperature of 1000 °C, which were mixed with varying percentages of glass waste, specifically at levels 10, 20, 30, 40, and 50 wt%. The recorded hardness values for these samples were 4.93 GPa for the 10% glass waste mixture, followed by 5.86 GPa for the 20 wt% glass waste mixture, 7.01 GPa for the 30 wt% mixture, 7.62 GPa for the 40 wt% mixture, finally reaching 7.89 GPa for the sample containing 50 wt% glass waste, as detailed in Table 4. The results show that the hardness is highly dependent on the amount of cullet in the samples. It was observed the increase in the cullet leads to enhance in hardness values. The maximum hardness value is 7.9 GPa with an increase in cullet up to 50 wt% and the minimum value is 4.8 for pure kaolin sample. These results are caused by the loss of flexibility following the addition of a cullet, which results in the dehydroxylation of clay and the loss of surface water or physiosorbed water, respectively. Additionally, the forceful separation of clay particles weakens the strength by breaking homogeneous contact^22^. The chemical and mineralogical makeup of the raw materials, the production temperature, the pace of heating, the firing interval, and the kiln atmosphere (oxidation or reduction) generally affect how strong the ceramics get. The mechanical qualities rise as the amount of cullet increases. When cullet is melted at high temperatures by reacting oxides, the silica content increases. This results in soda lime glass, which gives off hardness when cooled to room temperature. The quantity of free silica that is present tends to grow with cullet content, and when the temperature rises, it melts to produce a homogeneous, thick structure that significantly increases body strength^22^. Thus, using cullet improves ceramic materials’ strength at low temperatures without using high temperatures to get the same result, saving time, money, materials, and equipment life cycle. Additionally, at lower percentages of glass waste, specifically ranging from 0 to 20%, the hardness values exhibit a moderate increase. This trend indicates that the initial incorporation of glass waste plays a beneficial role in enhancing the densification process during the sintering phase^3,65^. The presence of glass waste is likely instrumental in facilitating the formation of a more compact microstructure within the ceramic material. By effectively filling voids and reducing porosity, glass waste contributes to a denser and more uniform structure. This reduction in porosity is particularly critical, as it directly influences the mechanical strength of the final product^65^. A denser microstructure not only improves hardness but also enhances overall durability, making it a vital factor in optimizing the performance characteristics of ceramics^3,65^.

As the percentage of glass waste increases, specifically from 30 to 50 wt%, the hardness values continue to rise significantly, ultimately reaching 7.89 GPa at 50 wt% of the cullet. This notable increase in hardness suggests that at these higher concentrations, the glass phase plays a vital role in enhancing inter-particle bonding and facilitating improved phase interactions within the Kaynite matrix as indicated by XRD analysis. The greater amount of glass waste contributes to a more cohesive and interconnected microstructure, which is essential for achieving superior mechanical properties^3,65,66^. Enhanced inter-particle bonding results in a more robust framework that can better withstand applied stresses, thereby leading to increased hardness. On the other hand, the development of a liquid phase during the firing process can significantly enhance the rearrangement and bonding of particles, ultimately leading to a denser and more resilient ceramic structure. This liquid phase acts as a bonding agent, allowing particles to move more freely and settle into a more compact arrangement, which is vital for achieving optimal mechanical properties^3,65–67^. However, it is important to recognize that while the addition of glass waste generally correlates with improved hardness, there are potential downsides associated with excessive amounts. Overly high concentrations of glass waste can introduce an abundance of glassy phases, which may result in increased brittleness or diminished toughness. These changes can compromise the structural integrity of the ceramic material, making it more susceptible to fracture under stress. Therefore, while incorporating glass waste can enhance hardness, careful consideration must be given to the balance of its concentration to avoid adverse effects on the overall durability and performance of the ceramic product.

### Studying the effect of incorporation of cobalt oxide and cupper oxide into kaolin-cullet mix

This section examines the impact of certain transition metal oxides (Co_3_O_4_ and CuO) on the optical, structural, and mechanical properties following sintering at 1000 °C using a mixture that contains 70 wt% kaolin and 30 wt% cullet.

#### Phase analysis after incorporation of transition metal oxide into kaolin-cullet mix

**Fig. 7:**
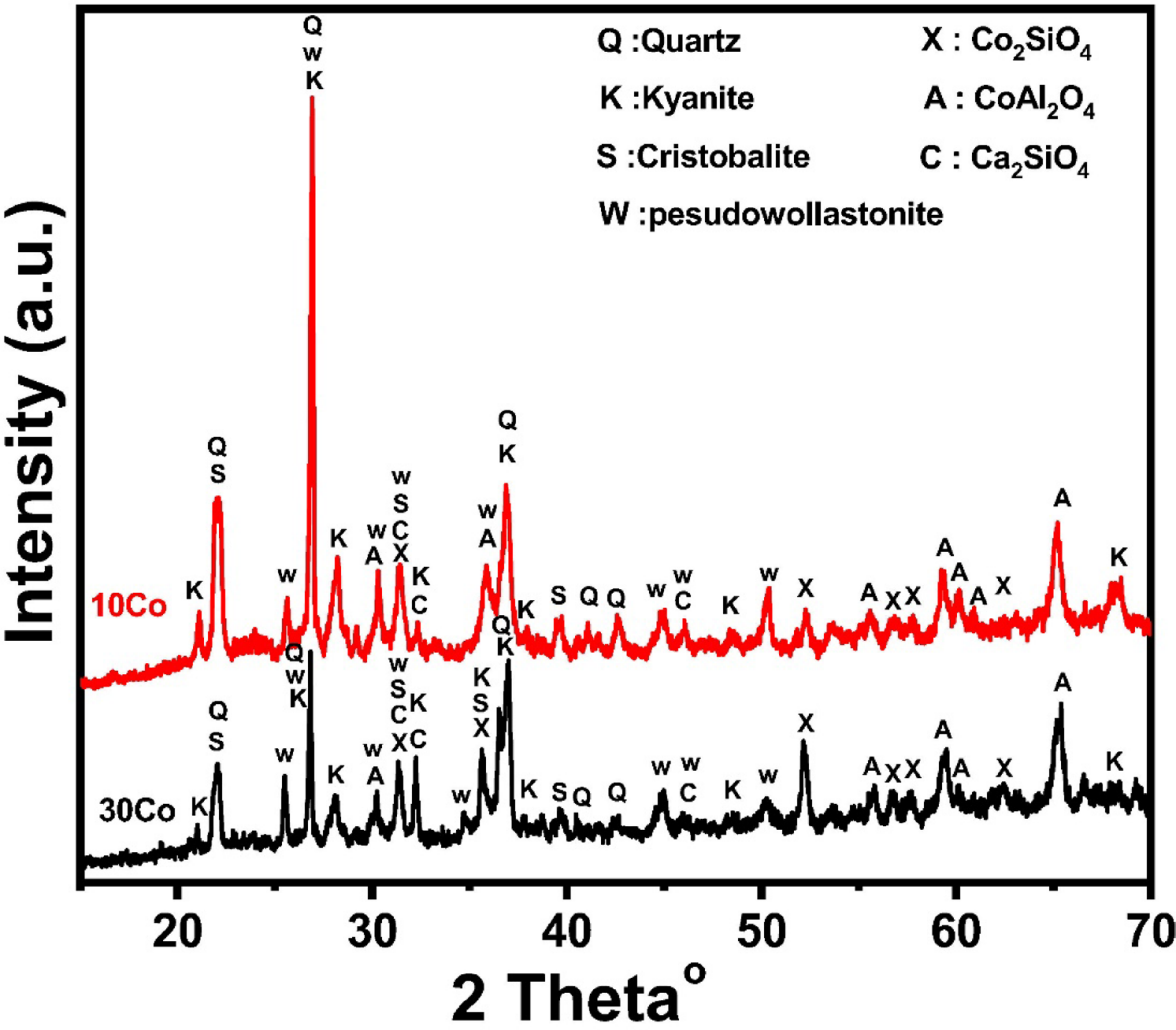
X-ray diffraction pattern of selected Co_3_O_4_ doped—samples.

Figure 7 shows the effect of adding Co_3_O_4_ to kaolin for a mixture of 70 wt% kaolin and 30 wt% cullet for 10Co and 30Co samples sintered at 1000 °C. Quartz (JCPD 01-086-1565) is the predominant phase in the sample with 10 weight percent Co_3_O_4_, with trace amounts of cobalt aluminate spinel (JCPD 01-82-2252), kyanite (JCPD01-087–1710), Ca_2_SiO_4_ (JCPD 01-86-0401), and cristobalite (JCPD 001-0424) also present. When the addition of Co_3_O_4_ is raised to 30 wt%, a decrease in cristobalite is also observed, along with some cobalt silicate, and calcium silicate phases alongside the major phases of cobalt aluminate and quartz^62^.

**Fig. 8:**
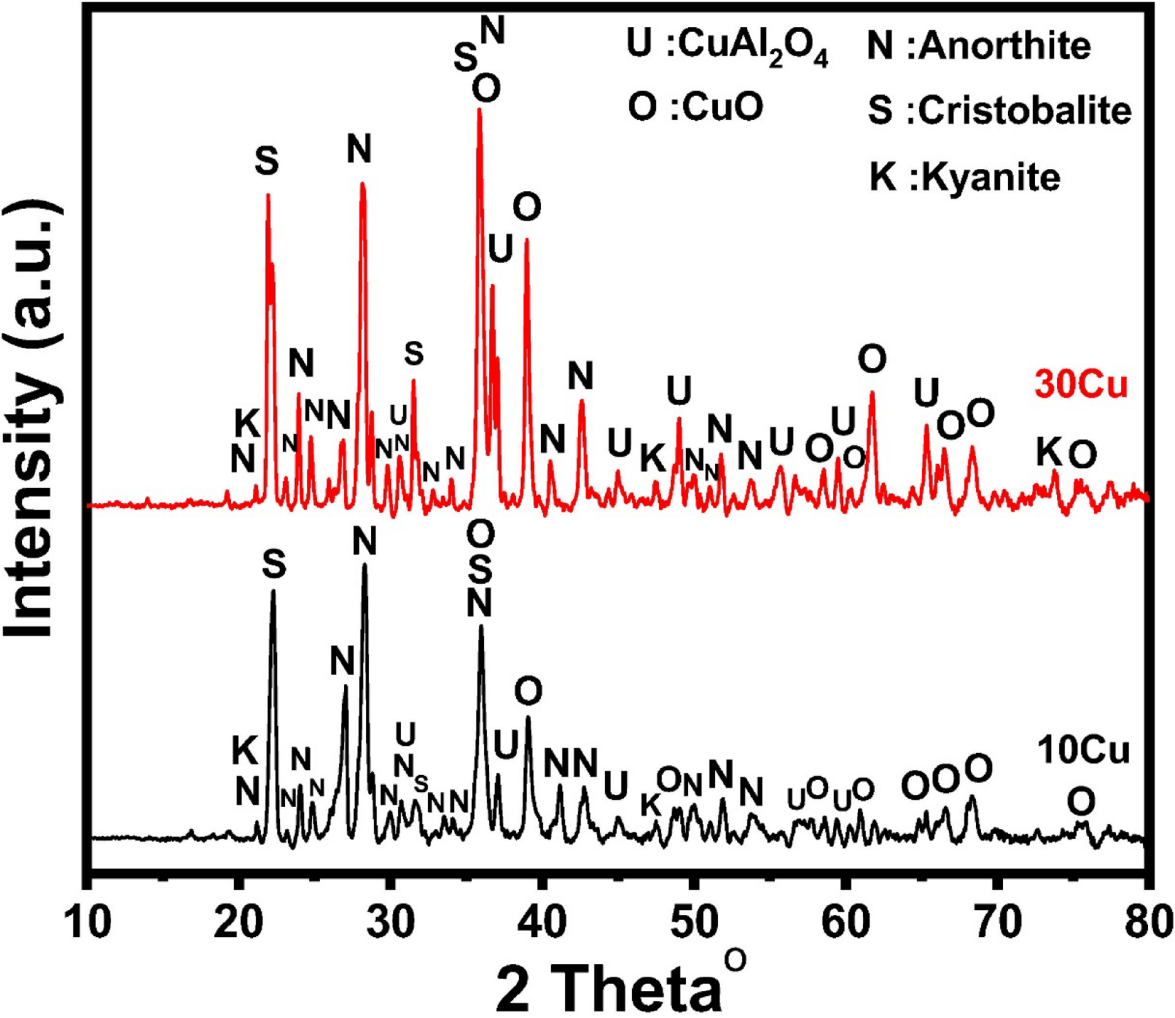
X-ray diffraction pattern of selected CuO doped—samples.

However, Fig. 8 shows the addition of CuO on 70 wt% Koalin-30 wt% cullet at the expense of kaolin. When 10 wt% CuO was added, it was found that the predominant phases formed were quartz and cristobalite, with trace amounts of CuO (JCPD 01-074-1021) and anorthite (JCPD01-085-1660), as well as CuAl_2_O_4_ spinel (JCPD01-073-1958). In addition to quartz and cristobalite, anorthite, spinel (CuAl_2_O_4_), and excess CuO oxide are seen when the levels of CuO additions are increased to 30 wt%^68^. Since these samples include more cristoballite than cobalt oxide, it’s probable that the primary kyanite that forms will interact with copper ions. After alumina and copper oxide react, kyanite is destroyed and copper aluminate is created. Amorphous SiO_2_ exudes and crystallizes as cristobalite as kyanite breaks down^47^.

#### Physical properties after incorporation of transition metal oxide into kaolin-cullet mix

**Fig. 9:**
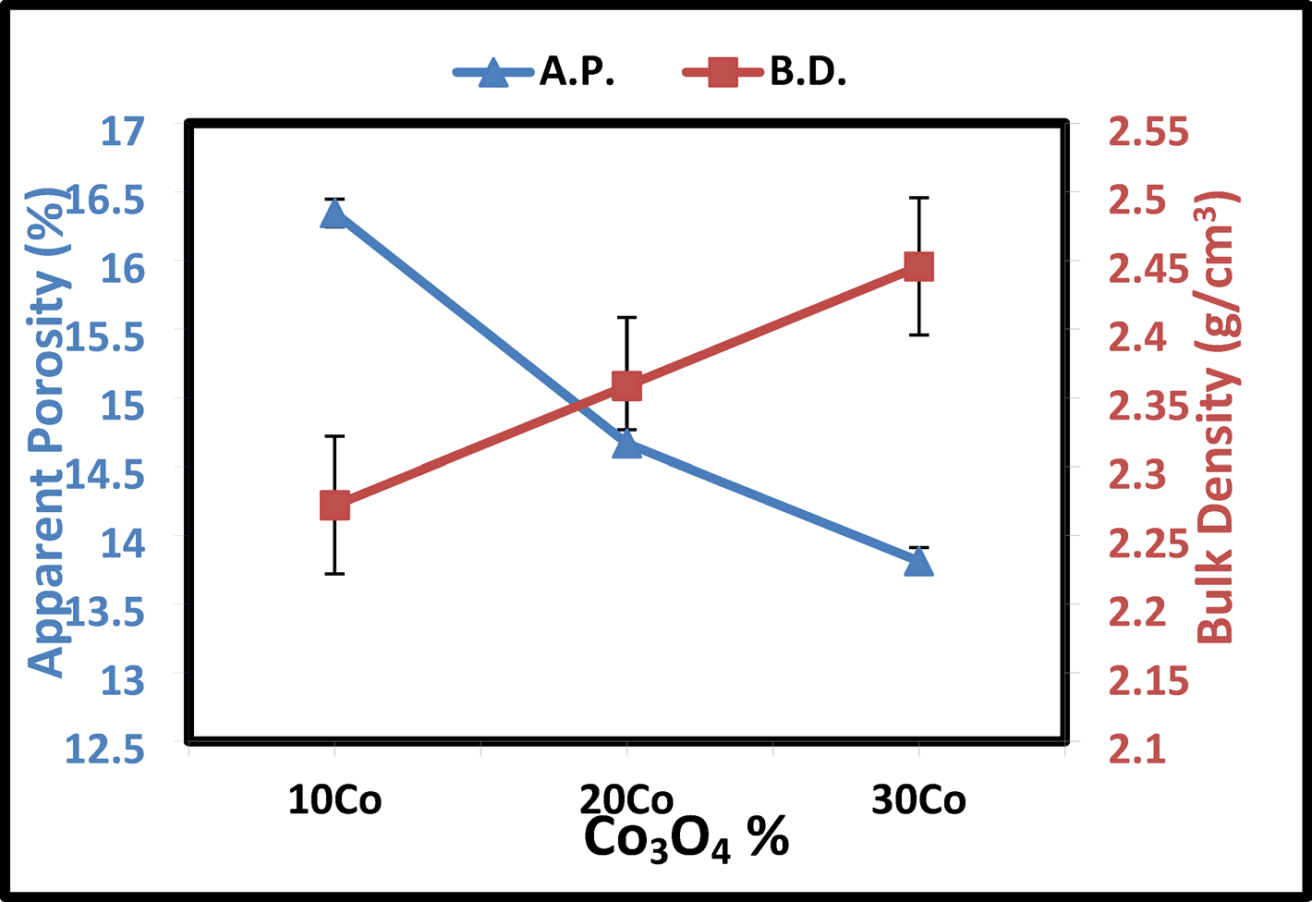
The apparent porosity and bulk density of Co_3_O_4_—doped samples sintered at 1000 °C.

**Fig. 10:**
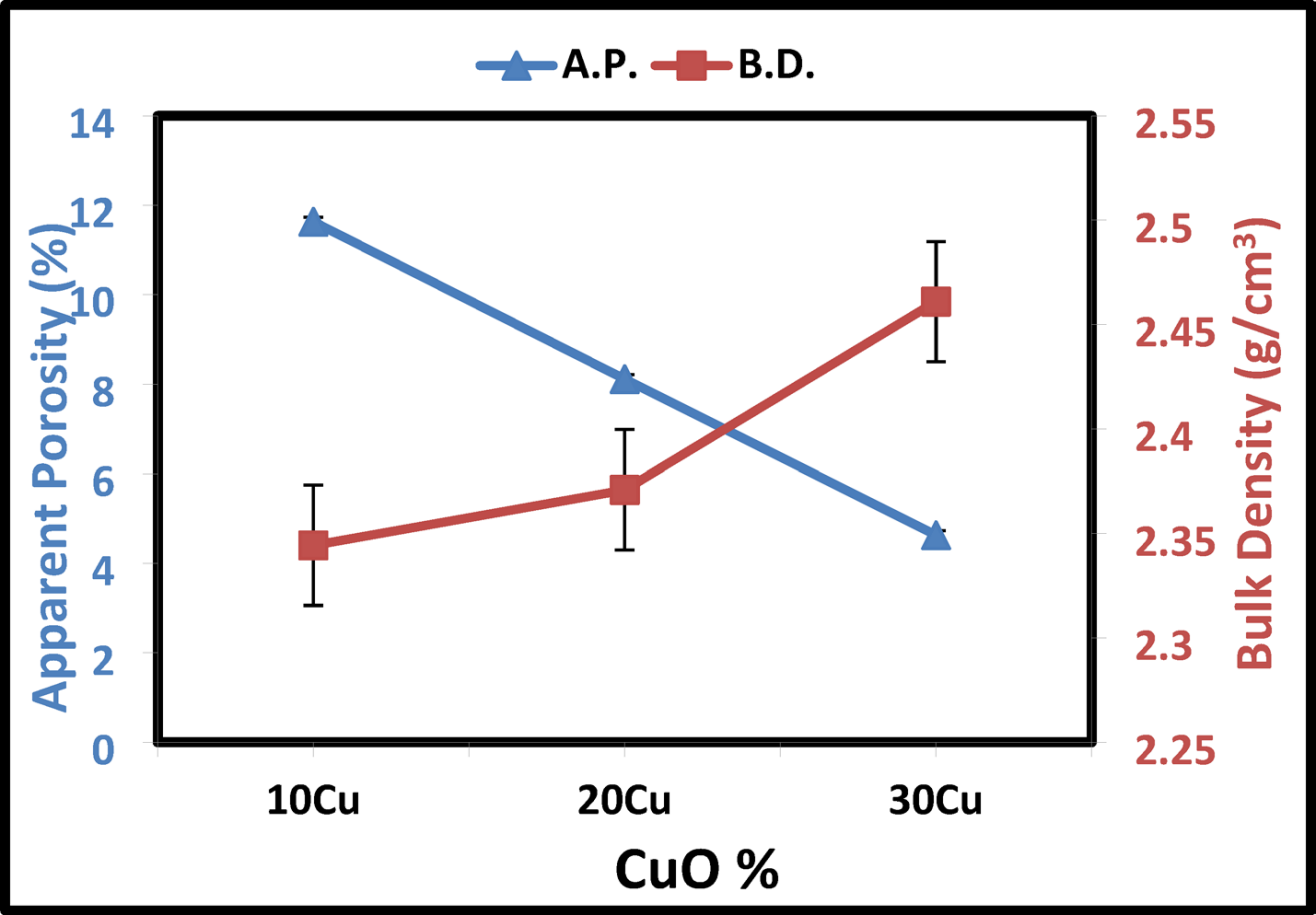
The apparent porosity and bulk density of CuO—doped samples sintered at 1000 °C.

Sintering is the process of solidifying particles at a temperature below the melting point. A verification method called viscous flow sintering is commonly employed for sintering in complex multi-phase systems like kaolin-cullet^69–71^. The physical properties in terms of bulk density and apparent porosity with the addition of Co_3_O_4_ and CuO are displayed in Figs. 9 and 10, respectively. When examining structural changes in materials, such as modifications to the geometrical configuration, coordination number, cross-linked density, interstitial space dimension, structural softening, and compactness, density is a helpful tool^71,72^. In samples containing cobalt oxide, it was demonstrated that porosity decreased as cobalt additions increased. The porosity reaches its lowest of 16.3 after adding 30 wt% Co_3_O_4_, and its greatest of 18.8% after adding 10 wt% Co_3_O_4_. The maximum bulk density of approximately 2.44 g/cm^3^ is reached after adding 30 wt% of Co_3_O_4_. Consequently, when a glassy phase is present, the presence of this Co_3_O_4_ facilitates the densification process. As so, it facilitates the dissolution of quartz and reduces the glassy phase’s viscosity. Consequently, when a glassy phase is present, the presence of this Co_3_O_4_ facilitates the densification process^69,70^. Additionally, it was observed that when the Co_3_O_4_ level increased, so did the samples’ densities. The lighter SiO_2_ (2.64 g/cm^3^) may be partially replaced by the heavier Co_3_O_4_ (6.11 g/cm^3^), which could account for this^73,74^.

As illustrated in Fig. 10, the bulk density of the investigated samples rose from 2.34 to 2.46 g/cm^3^ as a result of increasing the CuO concentration added to the kaolin-glass matrix at the expense of kaolin. When CuO is added, the apparent porosity drops from 11 to 4%, as shown in Fig. 10. There are two possible reasons for this: For starters when the CuO content is added to the glass matrix, the total atomic mass and molecular weight of the kaolin-cullet composition sample increase due to the high values of atomic mass (79.547) and molecular weight for CuO^75,76^.

Because molecular weight and density are closely related, increasing the glass composition sample’s CuO content increases its total density. When CuO is added to the glass matrix, the overall density of the kaolin and cullet composition sample increases, which could be the second explanation due to CuO has high density (6.3 g/cm^3^)^71,72,76^. When CuO is added, the porosity drops from 11 to 4% after heat treatment at 1000 °C. This is because CuO is commonly utilized as a metal nucleating agent in glass, where it is dispersed in colloidal particles to promote crystallization by inducing nucleation during a subsequent heat treatment^67^.

Because Cu ions have a larger molecular mass (63.5 gm/mol) than Co ions (58.933 g/mol), they typically have a higher density than Co ions. The range of its filling to the network gaps has expanded, nonetheless, due to Cu has larger ionic radius (0.073 nm) than Co has (0.072 nm). As a result, the molar volume drops more drastically for the glass with higher concentrations of Cu than for the glass with lower concentrations of Co^77^.

#### SEM analysis after incorporation of transition metal oxide into kaolin-cullet mix

**Fig. 11:**
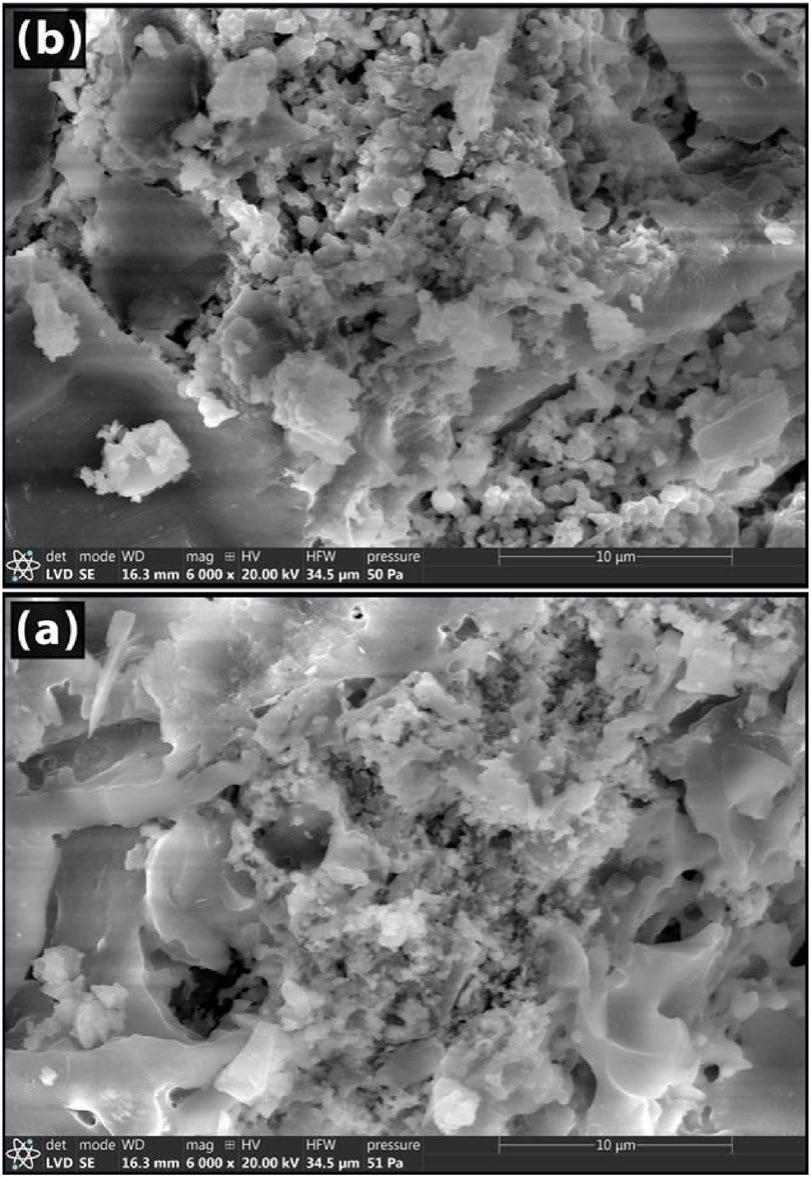
SEM images of Co_3_O_4_—doped samples sintered at 1000 °C.

**Fig. 12:**
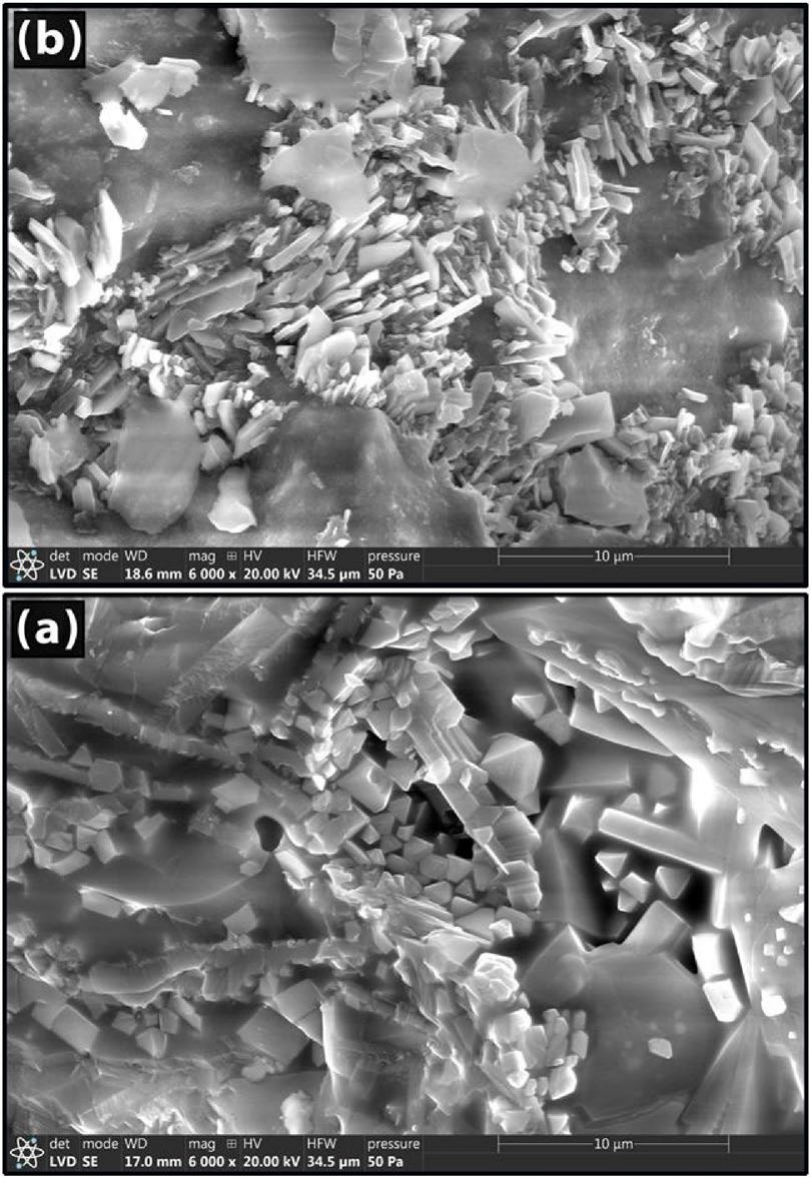
SEM images of Co_3_O_4_—doped samples sintered at 1000 °C.

SEM is used to evaluate the prepared products’ morphology and characterize them. The morphology or shape of the produced powders is ascertained using scanning electron microscopy. Figures 11 and 12 show the morphology following heat treatment at 1000 °C using 10 and 30 weight percent of Co_3_O_4_ or CuO. In the cobalt-containing samples (Fig. 11), the particles accumulate together and have an uneven shape. Some of these formations are very large, with many little particles compactly aggregated together, whereas other particles are also small aggregates, but they are connected in an irregular pattern. Additionally visible are tiny holes or pores that shrink as the cobalt content rises.

Figure 12 shows the SEM analysis for samples 10 and 30 Cu. Figure 12b ^78^ showed that the CuO particles had a roughly equiaxed form without any sharp edges. The calcinated CuAl_2_O_4_ was found to have a cubic structure. As seen in Fig. 12b ^79^, it is clear that copper oxide additions have encouraged the production of anorthites. Anorthite appears as a rod-like structure^80,81^. When comparing samples with Cu ions to those with Co ions, a greater quantity of glass phase is found at the grain boundaries^80,81^.

#### Hardness after incorporation of transition metal oxide into kaolin-cullet mix

Hardness values for samples incorporating cobalt oxide (Co_3_O_4_) or copper oxide (CuO) at concentrations of 10%, 20%, and 30 wt% sintered at 1000 °C are also presented in Table 4 and Fig. 6. The Co samples exhibited hardness values of 7.53 GPa, 7.93 GPa, and 8.22 GPa, respectively, while the Cu samples showed slightly lower values of 6.98 GPa, 7.31 GPa, and 7.87 GPa for the same concentrations. This observed progressive increase in hardness with the addition of glass waste suggests a positive correlation between the glass content and the mechanical properties of the ceramic material, highlighting the potential benefits of incorporating glass waste into ceramic formulations to enhance their durability and performance.

The incorporation of transition metal oxides, specifically cobalt oxide (Co_3_O_4_) and copper oxide (CuO), into the prepared ceramic samples at concentrations of 10%, 20%, and 30% has been shown to lead to a noticeable increase in microhardness values when fired at a temperature of 1000 °C, as depicted in Fig. 6. This progressive enhancement in hardness with higher concentrations of Co_3_O_4_ or CuO suggests that the addition of these metal oxides significantly aids the densification process during sintering, resulting in a more refined and compact microstructure. The hardness values obtained indicate that an optimal range of transition metal oxides not only improves inter-particle bonding but also fosters the formation of stable phases within the ceramic matrix, which is vital for overall material performance. However, it is important to note that while the hardness tends to increase more markedly with Co_3_O_4_ compared to CuO, potential trade-offs must be considered, particularly regarding brittleness that may develop at elevated concentrations. Microstructural analysis is likely to reveal finer grain sizes and reduced porosity, both of which are essential for achieving enhanced mechanical properties.

Building on the previous discussion, several primary factors that affect the hardness can be summarized^65,82^; First and foremost is the sintering temperature; higher temperatures facilitate increased crystallization and density, which enhance hardness. For example, kyanite transforms into more stable phases when subjected to elevated temperatures, typically between 1050 and 1200 °C, thereby improving its mechanical properties. Another important factor is phase composition; the presence of various crystalline phases within the ceramic matrix can significantly influence microhardness. The formation of specific phases during sintering can enhance both hardness and overall mechanical performance in kyanite ceramics than in anorthite ceramics. In contrast to samples including cobalt, ceramics containing anorthite promote the production of a large amount of glassy phase, which lowers the hardness value^80,81^. When taken as a whole, these elements emphasize how important it is to optimize sintering conditions and material compositions to produce ceramics with exceptional mechanical properties^80–82^.

#### Diffuse reflectance spectra analysis

**Fig. 13:**
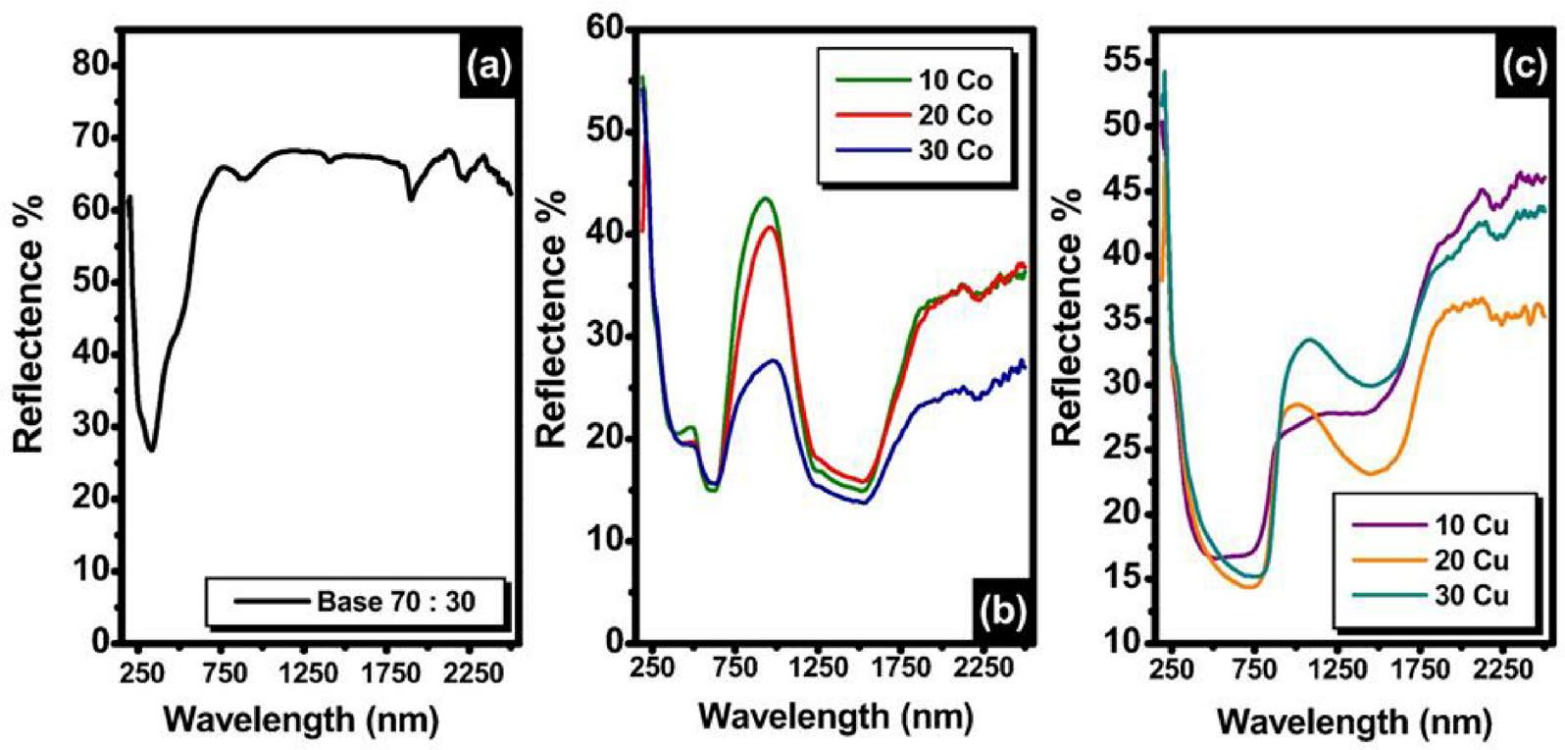
UV–Visible-NIR of the undoped and Co_3_O_4_ or CuO—doped samples.

**Fig. 14:**
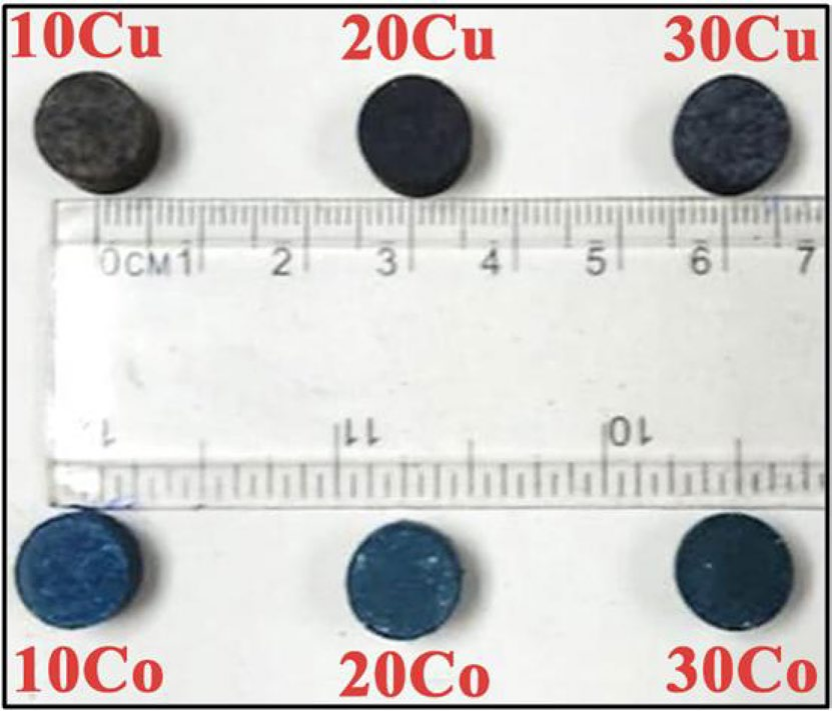
Photographic image of Co_3_O_4_ and CuO—doped Samples.

UV–visible-NIR reflectance investigations are necessary to make sure the manufactured material can be used in the specified applications. The variations in the UV–visible NIR spectra following the addition of varying concentrations of Co_3_O_4_ or CuO in 70 weight percent kaolin-30 weight percent cullet are shown in Fig. 13. The digital pictures of the 10 Co, 20 Co, 30 Co, 10, Cu, 20 Cu, and 30 Cu samples that illustrate the color differences are included in the inset (Fig. 14). After adding CuO, we can observe that the hue shifts from brown to dark brown and then to black. It is determined that samples with varying concentrations of Co_3_O_4_ undergo a color shift from blue to dark blue. As a result, the color variation may serve as direct proof of how the sintering temperature and transition metal concentration affect the individual samples.

As shown in Fig. 13b Co-containing materials show a high reflectance band in the near-infrared (NIR) region at approximately 800–1100 nm, as well as a visible band with a maximum at approximately 450–572 nm. The Co^2+^ (3d electronic configuration) A (F) → T (P) electronic transition in tetrahedral coordination^83^ is associated with the reflectance band at around 450–572 nm, which is equivalent to the energy, v (cm). The A(F) → T(F) transition of Co^2+^ cations in tetrahedral coordination^15,16,29^ is responsible for the observed broad reflectance band at around 800–1100 nm, which is equivalent to the energy, ν (cm^−1^)^83^.

The presence of Co^2+^ ions is the reason why the color samples changed from blue to dark blue. In actuality, the presence of Co^2+^ ions results in color centers inside glasses that have a characteristic visual reflectance band, giving them both pink and bluish hues. The arrangement of the Co^2+^ ions (transformation from tetrahedral (Td) to octahedral (Oh)) determines how vivid the blue hue is. As more CoO was added to the samples, the hue of the Co-doped samples shifted from light to dark blue, indicating an increase in the absorption band and a decrease in reflectance band intensities. Beer-Lambert-Bouguer’s rule and behaviors described in earlier publications^83^ are very consistent with this.

Figure 13c shows that the CuO content affects the band’s height, width, and area. Figure 13c shows the relationship between reflectance and wavelength range (λ = 200–2500 nm) for the samples under investigation. Without any notable features, it was found that the reflectance bands covered spectra in the NIR region (800–1250 nm). The tiny energy gaps are what this signifies. Furthermore, it is evident that as the CuO content drops, the reflectance band’s height nearly drops. The concentration of Cu^2+^ ions, which may function as a network modulator, is responsible for this behavior. Copper can exist as Cu^2+^ and Cu^+1^ ions^84^. Every sample that contains Cu exhibits a reflectance of less than 40% in the 230–2500 nm wavelength range. This material has a low reflectance value allowing it to be employed as an antireflection coating in the production of solar cells. Tangcharoen et al.^85^ explained that CuAl_2_O_4_ showed distinct absorption characteristics in both the VIS and NIR areas depending on the sintering temperature and that this behavior is caused by the sintering of samples at a high temperature (1000 °C). They deduced from their electron paramagnetic resonance (EPR) spectra that this phenomenon was caused by the Cu^2+^/Cu^+^ mixed valencies and defects such as oxygen vacancies in the anion network of the sample that was synthesized at a low temperature. The increase in absorbance strength at about 780 nm is influenced by the intra-atomic transition (3d^10^ → 4s^0^) in these monovalent copper cations (Cu^+^). Conversely, the oxidation process Cu^+^  → Cu^2+^  + e^–^ is the result of calcination at 1000 °C. The shift of the absorption band from around 800 to 1250 nm may be caused by the inter-atomic d–d charge transfer between divalent and monovalent copper cations (Cu^2+^/Cu^+^ inter-valency transitions), which are directly impacted by this rise in Cu^2+^^85^.

#### Compressive strength

**Fig. 15:**
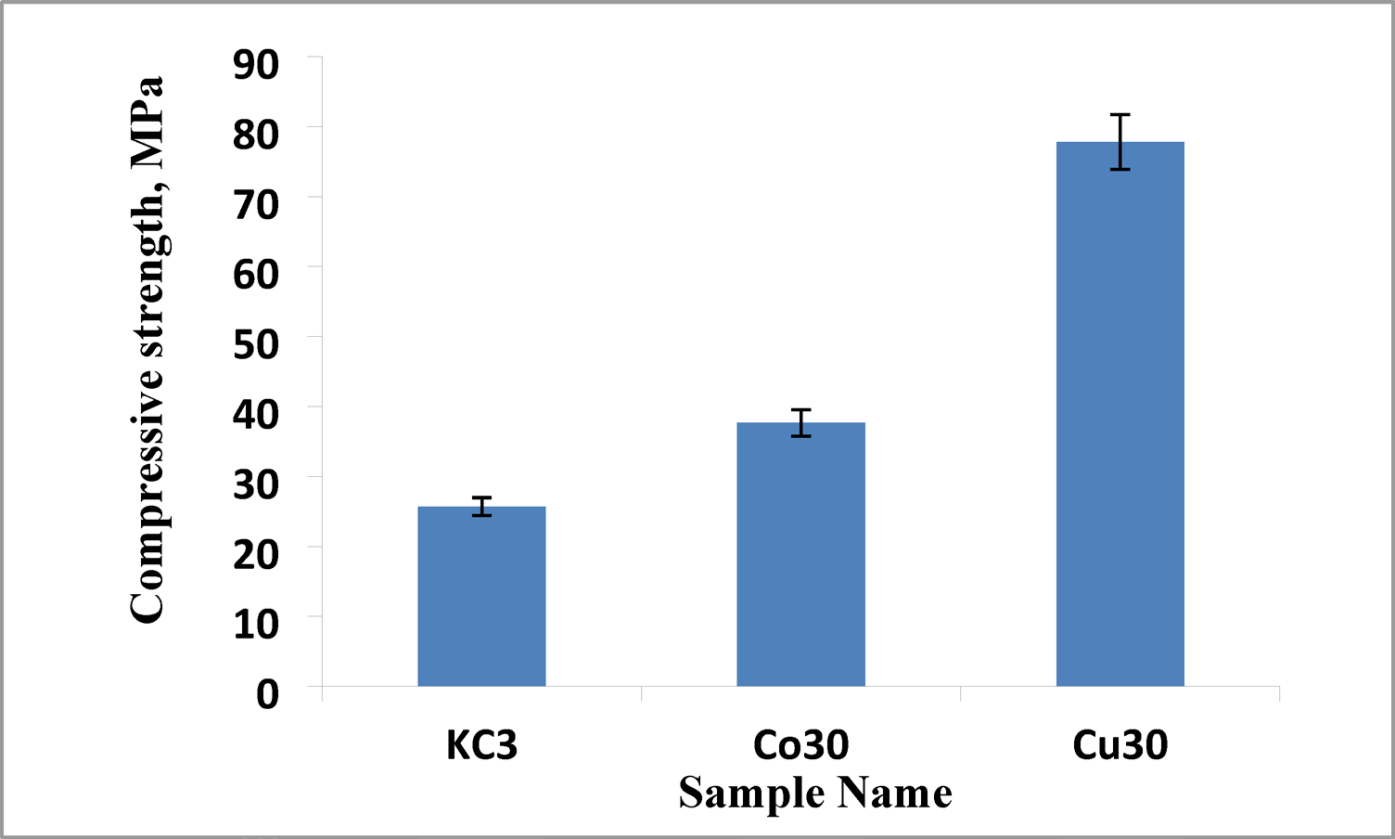
The compressive strength of selected samples sintered at 1000 °C.

Figure 15 displays the compressive strength of the KC3, Co30, and Cu30 samples that were sintered at 1000 °C. Compared to the KC3 sample (25.3 MPa) or the Co30 sample (37.65 MPa), the Cu30 sample’s compressive strength is higher at 77.8 MPa. The mechanical properties of materials are often determined by a number of factors, including porosity, sintering temperature, and material type and composition^86^. The development of a dense strut between grains and/or a reduction in porosity are known to be associated with the strength increase^87,88^. The increase in the mechanical strength is in agreement with the decrease in the apparent porosity, shown in Figs. 1 and 9. The sample is destroyed because the inner porosity of the samples concentrates stress during the tests^89^.

## Conclusion

A series of ceramic materials were produced by incorporating up to 50% glass cullet waste into kaolin, with and without the addition of Co_3_O_4_ or CuO at different sintering temperatures, to optimize and investigate their effects on the physical, structural, and optical properties of the ceramics. XRD analysis identified kyanite and anorthite at 1000 °C, and sillimanite and anorthite at 1200 °C. Increasing the cullet content to 50 wt% reduced apparent porosity and increased bulk density, reaching 13% porosity and 2.75 g/cm^3^ density after sintering at 1000 °C. However, temperatures above 1000 °C resulted in reduced densification when 30 wt% of cullet was used. Hardness values peaked at 7.8 GPa for the 50 wt% cullet composition after heat treatment at 1000 °C. The addition of Co_3_O_4_ or CuO to a 70 wt% kaolin and 30 wt% cullet mixture improved densification. Specifically, 30 wt% Co_3_O_4_ resulted in a bulk density of 2.44 g/cm^3^ and 13% porosity, while 30 wt% CuO gave a density of 2.46 g/cm^3^ and porosity of 4%. Cobalt-based ceramics showed higher hardness (8.22 GPa) compared to copper-based ones (7.87 GPa). Optical analysis revealed that CuO-containing samples had reflectance below 40%, suggesting their potential use in solar energy technologies, such as solar panels or thermal collectors, where light absorption and reflection are necessary for efficiency.

## Data Availability

The datasets generated and/or analyzed during the current study are not publicly available because they are private, but are available from the corresponding author on reasonable request.
